# Metabolic changes in dorsal root ganglia of newborn Wistar rats following LPS exposure to understand mechanisms of critical illness polyneuropathy

**DOI:** 10.14814/phy2.70633

**Published:** 2025-11-02

**Authors:** Amandine Szczesnowski, Christelle Le Gall‐Ianotto, Marie‐Dominique Jezequel, Michaël Théron, Karelle Léon, Karine Pichavant‐Rafini

**Affiliations:** ^1^ ORPHY Univ Brest Brest France; ^2^ Department of Dermatology University Hospital Brest France; ^3^ LIEN Univ Brest Brest France

**Keywords:** dorsal root ganglia, glycolysis, lipopolysaccharide, oxidative phosphorylation

## Abstract

Sepsis is a public health issue, associated with complications as critical illness polyneuropathy (CIP). This study investigates how mitochondrial dysfunction could contribute to CIP by examining metabolic changes in dorsal root ganglia (DRG) culture from newborn Wistar rats exposed to different concentrations of lipopolysaccharide (5, 10, 50, and 100 μg/mL for 24 or 48 h). Cell viability was assessed using the MTS assay, gene expression related to inflammation and mitochondrial function was analyzed by Real‐Time PCR. IL‐6 levels of supernatants were measured by ELISA and energetic metabolism was evaluated with the Seahorse MitoStress kit. Exposure to LPS at varying concentrations mainly did not affect cell viability, except at 10 μg/mL for 48 h, where a 7.5% increase was noted. Gene expression analysis showed trends in SOD2 and Fis1, with significant increases in IL‐6 and LIF transcripts at 5 and 10 μg/mL. IL‐6 release was significant at 50 and 100 μg/mL of LPS. Mitochondrial respiration and glycolytic metabolism exhibited significant changes in oxygen consumption rate and extracellular acidification, particularly at higher LPS concentrations. The findings suggest that LPS induces an inflammatory environment which led to metabolic disturbances in DRG cells, with adaptive responses at 10 and 50 μg/mL of LPS.

## 1 INTRODUCTION

Since 2016, sepsis is defined as a life‐threatening organ dysfunction caused by a dysregulated host response to infection (Singer et al., 2016). It is a critical illness and a major public health issue worldwide. In 2017, there were approximately 49 million cases, resulting in 11 million deaths–accounting for 20% of worldwide mortality (Rudd et al., 2020). Many patients developed complications. Among them, a particularly harmful complication is intensive care unit–acquired weakness that often occurs. Indeed up to 65% of septic patients developed this condition (Piva et al., 2023). It may be the result of three different pathologies including the critical illness polyneuropathy (CIP). Development of CIP is associated with increased mortality, prolonged times of respiratory assistance, and longer rehabilitation periods (Garnacho‐Montero et al., 2001). CIP is defined as a sensory–motor axonal polyneuropathy (Piva et al., 2023). It manifests by muscular weakness, a decrease in tendon reflexes and a loss of sensitivity to pain, temperature and vibration (Hermans et al., 2008; Hermans & Berghe, 2015).

The physiopathology of CIP is not fully understood. However, it involves complex interactions, including alterations in microvascular circulation, abnormalities in nerve conduction, a pro‐inflammatory environment, and metabolic and bioenergetic dysfunctions (Fenzi et al., 2003). The nerve conduction studies indicate a reduction in the amplitude of sensory nerve action potentials and suggest sensory peripheral nerve damage (Piva et al., 2023). An impairment of small nerve fibers in sepsis may play a major pathophysiological role in neuropathic pain syndromes, that often appear after recovery from critical illness (Latronico et al., 2005). Sensory neurons are altered by sepsis but are also involved in many inflammatory conditions. That is why they can be considered targets for the modulation of the inflammatory response. Neurons are highly energy‐consuming cells and sensitive to mitochondrial dysfunctions. Their metabolism is only aerobic and oxidative. They use mainly oxidative phosphorylation, Krebs cycle and glycolysis pathway. Besides, neurons require a constant import of glucose to function effectively; it is their primary energetic substrate. Any perturbations in the mitochondrial machinery may induce severe mitochondrial dysfunctions and alter metabolism as well as generate some oxidative stress which are both deleterious for neurons and cerebral functions (Trigo et al., 2022).

Given the key role of mitochondria, the understanding and characterization of energetic metabolism of neuronal cells seem needed.

To explore the metabolic changes associated with CIP due to pathogen exposure, we used a mixed primary culture model containing sensory neurons and support cells derived from dorsal root ganglia (DRG), exposed to various concentrations of LPS to simulate clinical conditions encountered in sepsis. This approach enhances the representativeness of our findings and deepens our understanding of the underlying mechanisms, considering the variability in disease severity.

Our objectives are to (1) investigate mitochondrial gene expression and the balance between oxidative and glycolytic metabolism in DRG cells during inflammation, and (2) determine the primary energy substrate utilized by these cells in an inflammatory environment. This research aims to enhance the intricate relationship between mitochondrial dysfunction and energy metabolism in the context of sepsis.

## 2 MATERIALS AND METHODS

### 2.1 DRG cells extraction and cell culture

The DRG‐specific cell types in DRG primary cultures are neurons (about 10%), satellite glial cells (about 80%) and macrophages (about 2%) (Neuroscience 394: 1–13, 2018). They were obtained from newborn Wistar rats (female and male, 2–5 days old) and were enzymatically dissociated in 250 μg/mL collagenase IV solution (C1889, Sigma‐Aldrich) (dissolved in DMEM supplemented with Normocin™ 500X; ant‐nr −2, Invivogen) for 40 min at 37°C. Mechanical dissociation was performed with multiple passages through pipette tips. Two cycles of mechanical dissociation followed by centrifugation at 240*g* for 5 min were performed. Then, DRG cells were cultured in a 96‐well plate precoated with 5 μg/mL poly‐L‐lysine (P4707, Sigma‐Aldrich) with a seeding ratio of one neonatal rat per 20 wells. Cells were cultured in a mix of DMEM and DMEM/F12 medium (50% of Ham‐F12 and 50% of DMEM; Gibco, Thermo Fisher) supplemented with Normocin™ 500X, NGF‐β (100 ng/mL; N1428, Sigma‐Aldrich), insulin (4 μg/mL; I6634, Sigma‐Aldrich), hydrocortisone (10 ng/mL; H0135, Sigma‐Aldrich), B27 (50X; 17504044, Gibco, Life Technologies) and BDNF (25 ng/mL; sc4554, Santa Cruz Biotechnology) and maintained at 37°C in a humidified atmosphere of 95% air and 5% CO_2_ for 6 days. The medium was changed every 3 days until experimental use.

### 2.2 Exposure to LPS and viability assay

After seeding, DRG cells were incubated for 6 days to allow the development of the network related to the growth of satellite glial cells which will start to surround neurons after seeding. Then, the DRG cells were exposed for 48 h to 0, 5, 10, 50, and 100 μg/mL of LPS (00‐4976, Thermofischer Scientific) diluted in a mix of DMEM/DMEM‐Ham‐F12 media supplemented as previously described in Section 2.1.

Cell viability was evaluated using the cell titer 96 Aqueous MTS reagent (CellTiter 96® AQ_ueous_ One Solution Cell Proliferation Assay (MTS), Ref G3582, Promega®) which is a colorimetric assay. Briefly, 20 μL of the reagent was added per 100 μL of supernatant. Optical density (OD) at 450 nm was measured using a spectrophotometer at 24 and 48 h after LPS exposure. OD measurements were reported relative to the control group, which was set at 100%.

### 2.3 Gene expression

#### 2.3.1 RNA isolation for RT‐PCR

Total RNA was extracted from cell lysates based on the instructions provided by the manufacturer with RNeasy® mini kit from QIAGEN RNeasy® (RNeasy Plus Mini Kit, 74314, Qiagen). Briefly, ethanol was added to the lysates and then transferred to the RNeasy column for centrifugation (15 s, 8000 g, room temperature). 700 μL of buffer RW1 was added and then centrifuged (15 s, 8000 g, room temperature). Subsequently, two washes were performed using the wash buffer (RPE) provided in the kit. Finally, RNA was eluted in 30 μL of DNase/RNase free water and collected after centrifugation (1 min, 8000 g, room temperature) and stored at −80°C. The concentration of total RNA was determined using the SimpliNano™ spectrophotometer (29‐0617‐12, GE Healthcare Life Sciences).

#### 2.3.2 Quantification of gene expression by real‐time reverse transcriptase‐PCR (RT‐PCR)

RNA samples containing 200 ng of RNA each were reverse transcribed into complementary DNA (cDNA) using commercially available cDNA synthesis kits (4368814, Applied Biosystems™). The reverse transcription was carried out according to the following program: 1 cycle at 22°C for 5 min, 1 cycle at 42°C for 30 min, 1 cycle at 85°C for 5 min, ending at 4°C. The obtained cDNA was diluted 1:2 in DNase/RNase‐free water and stored at −20°C until analysis.

Analyses were performed using the 7500 Fast Real‐Time PCR system (Applied Biosystems, Thermo Fisher Scientific). Amplification of target genes was conducted using specific primers from Eurogentec (Table 1), and quantification was performed by incorporating SYBR® Green (PB20.11‐05, Eurobio). A denaturation step at 95°C was conducted for 2 min, followed by 50 amplification cycles (denaturation: 5 s at 95°C; hybridization/extension: 30 s at 60°C). Each gene (*IL‐6*, *LIF*, *SOD2*, *mt‐ND1*, *Sirt3*, *Fis1*, *OPA1*, *Mfn1*, *Mfn2*, *Drp1*, *mt‐Cox2*, *mt‐Cox4i1*, *mt‐ATP6*, *β*‐*actin*) was amplified in duplicate. Data were normalized using β‐actin as a reference gene (Table 1). This choice was supported by the absence of significant differences in β‐actin RNA levels between the different experimental groups (*p*‐value >0.05). Data analysis was performed by comparing the relative expression of mRNA using the 2^−ΔΔCt^ method (Livak & Schmittgen, 2001).

**TABLE 1:** Primers sequences (Eurogentec) used for real‐time RT‐PCR analysis.

Target gene	Primer sequence (5′–3′)	Reverse sequence (5′–3′)	Accession number	Data base
*IL‐6*	CTCAGGGAGATCTTGGAAATG	CCAGTTTGGAAGCATCCATCA	NM_012589.2	134
*LIF*	CAGTGCCAATGCCCTCTTTA	GCATGGAAAGGTGGGAAATC	NM_022196.3	111
*SOD2*	TGGCTTGGCTTCAATAAGGAG	AAGATAGTAAGCGTGCTCCCA	NM_017051.2	129
*mt‐ND1*	CGCCTGACCAATAGCCATAAT	TTCGACGTTAAAGCCTGAGAC	ENSRNOT00000047550.4	112
*Sirt 3*	CTCATGGGTCCTTTGTATCAG	TCAGGTTTCACAACGCCAGTA	NM_001106313.2	130
*Fis1*	ACGCCTGCCGTTACTTCTTC	GCAACCCTGCAATCCTTCAC	XM_006249122.3	108
*OPA 1*	GGCACTTCAAGGTCGTCTCA	CACTGCTCTTGGGTCCGATT	NM_133585.3	108
*Mfn 1*	ATCTGGTGGAGATACAGGGCT	TCCCACAGCATTGCGTTGAT	NM_138976.1	136
*Mfn 2*	GCTCAGTCGGTTGGAAGTCA	GAAAGGAGTGCCTGCCTGAT	NM_130894.4	108
*Drp 1*	AGGTTGCCCGTGACAAATGA	CACAGGCATCAGCAAAGTCG	NM_053655.3	94
*mt‐Cox2*	AATCTCATCCGAAGACGTCCT	GTCACTGTAGCTTGGTTTAGG	ENSRNOT00000043693.3	96
*mt‐Cox4i1*	CCTGAAGGAGAAGGAGAAGG	ACTCATTGGTGCCCTTGTTCA	NM_017202.1	116
*mt‐ATP6*	CCTATGAGCAGGAGCCGTAA	TGGGAATTAGGGAGATGGGG	ENSRNOT00000046108.3	98
*β‐actin*	CTACAATGAGCTGCGTGTGG	GGATGGCTACGTACATGGCT	NM_031144.3	140

### 2.4 Elisa assay

Duoset™ Elisa kits (DY506, Bio‐Techne) were used to determine the levels of IL‐6 in the supernatants of DRG cells exposed for 48 h at 0, 5, 10, 50, and 100 μg/mL of LPS according to the manufacturer's instructions. Absorbance was measured at 450 nm using a microplate reader.

### 2.5 Mitochondrial respiration and energetic metabolism

Mitochondrial respiration was evaluated using the Seahorse MitoStress kit (Agilent, Cat. No. 103010‐100) following the manufacturer's instructions. Cells extracted from DRG were seeded in Seahorse assay microplates (96 wells) with growth medium described previously and incubated for 6 days to allow the development of the neural network. The day before the assay, an XF cartridge was hydrated with XF calibrant (Agilent, Cat. No. 103059‐000) and incubated overnight at 37°C in a non‐CO_2_ incubator. Prior to the assay, the culture media were changed to 180 μL/well of XF DMEM supplemented with 10 mM glucose, 2 mM glutamine, and 1 mM pyruvate (Agilent, Cat. Nos. 103575‐100, 103577‐100, 103579‐100, 103578‐100) and incubated for 1 h at 37°C (without CO_2_). The oxygen consumption rate (OCR) and extracellular acidification rate (ECAR) were measured using the XFe96 Seahorse Bioanalyzer (Agilent Technologies, Santa Clara, CA, USA). The final concentrations of injected compounds were 1 μM oligomycin, 5 μM carbonyl cyanide‐4 (trifluoromethoxy)phenylhydrazone (FCCP), and 1 μM rotenone + antimycin A (Agilent, Cat. No. 103010‐100). From the values obtained, mitochondrial respiration and energetic metabolism were measured and calculated on DRG cells and more particularly basal OCR, OCR related to ATP production, maximal OCR, coupling efficiency, proton leak, reserve capacity, basal extracellular acidification, glycolytic capacity, glycolytic reserve and mitochondrial ATP/Glycolytic ATP ratio. Data were reported relative to the control group, which was set at 100%.

The utilization of metabolic substrates by the mitochondria was determined using the Seahorse XF Substrate Oxidation Stress Test Kits (Agilent, Cat. Nos. 103672‐100, 103673‐100, 103674‐100). Various metabolic pathway inhibitors were used (final concentrations): Etomoxir (4 μM; irreversibly blocks CPT1a, thereby inhibiting the fatty acid pathway; Agilent, Cat. No. 103672‐100), UK5099 (2 μM; inhibits the mitochondrial pyruvate transporter and prevents glycolysis; Agilent, Cat. No. 103673‐100) and BPTES (3 μM; inhibits glutaminase 1 and blocks the amino acid pathway; Agilent, Cat. No. 103674‐100).

### 2.6 Statistical analysis

Statistical analyses were performed using Graph prism 9.0.2 software. Results are presented as mean ± standard deviation (SD).

After checking for normality using the Shapiro–Wilk test and verifying homogeneity of variances, a non‐parametric Kruskal–Wallis test followed by Dunn's post hoc test was applied for comparisons among more than two conditions. For comparisons between two conditions, either Student's *t*‐test or the Mann–Whitney test was used, depending on data distribution. Differences were considered significant for a *p*‐value < 0.05.

Each experimental condition included 3–12 biological replicates (as indicated in the figure's legend), each performed with 2–5 technical replicates.

## 3 RESULTS

### 3.1 Cell viability

**FIGURE 1:**
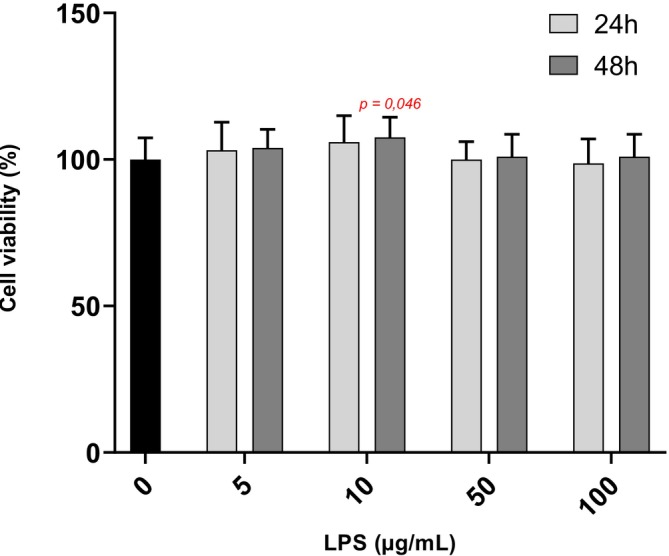
Viability of DRG cells by MTS assay, after 24 or 48 h of exposure to 0 (*n* = 12 biological replica); 5 (*n* = 4 biological replica); 10 (*n* = 4 biological replica); 50 (*n* = 6 biological replica); 100 (*n* = 3 biological replica) μg/mL of LPS. Values are represented as means ± SD. The Kruskal–Wallis test followed by Dunn's post hoc test was used to compare the effects of each LPS concentration with the control at 48 h. *p*‐values <0.05 are displayed in red on the graphs.

For all experimental conditions, except for exposure to 10 μg/mL of LPS for 48 h, no significant changes were observed in cell viability compared to the control group. However, after 48 h exposure of DRG cells at 10 μg/mL of LPS, an increase of 7.5 ± 2.3% in cell viability was observed compared to control cells (*p* = 0.046) (Figure 1).

### 3.2 Gene expression

**FIGURE 2:**
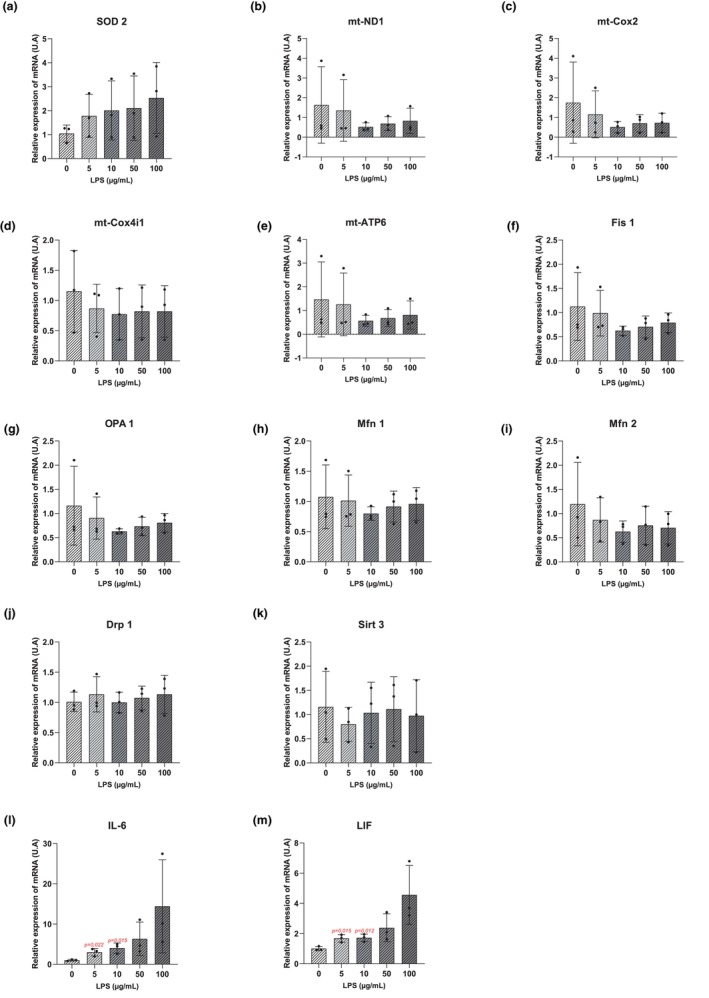
Relative expression of mRNA (arbitrary units (U.A)) in DRG cells exposed to 5, 10, 50 and 100 μg/mL of LPS for 48 h. Expression was normalized to β‐actin mRNA. The analysis 13 genes (Figure 2a–m) was performed using the ΔΔCT method for the groups: LPS 0 μg/mL (*n* = 3 biological replica); LPS 5 μg/mL (*n* = 3 biological replica); LPS 10 μg/mL (*n* = 3 biological replica); LPS 100 μg/mL (*n* = 3 biological replica). Results are presented as means ± SD. A Student's *t*‐test was used to compare each LPS concentration with the control condition. *p*‐values <0.05 are displayed in red on the graphs.

All experimental conditions did not lead to significant changes in the expression of *SOD2*, *mt‐ND1*, *mt‐Cox2*, *mt‐Cox4i1*, *mt‐ATP6*, *Fis1*, *OPA1*, *Mfn1*, *Mfn2*, *Drp1* and *Sirt*3 transcripts compared to the control group (Figure 2a–k). However, some trends may be described as an increase in mRNA levels of *SOD2* and an underexpression of *Fis 1*, *OPA 1*, *Mfn 2*, *mt‐ND1*, *mt‐Cox2*, *mt‐ATP6* transcripts compared to the control cells (Figure 2l,m). The lack of significant changes may be the result of the high variability of the data.

Nevertheless, the exposure to 5 and 10 μg/mL of LPS for 48 h induced overexpression of IL‐6 and LIF transcripts compared to the control group. Indeed, a 48 h exposure to 5 μg/mL of LPS increased 4 ± 0.5 times. IL‐6 transcript expression (*p* = 0.022) and 1.7 ± 0.1 times LIF transcript expression (*p* = 0.015). Exposure to 10 μg/mL of LPS resulted in a 5 ± 0.7 times increase in IL‐6 transcript expression (*p* = 0.015) and a 1.7 ± 0.1 times increase in LIF transcript expression (*p* = 0.012), compared to the control group (Figure 2l,m).

### 3.3 Quantification of IL‐6 release

**FIGURE 3:**
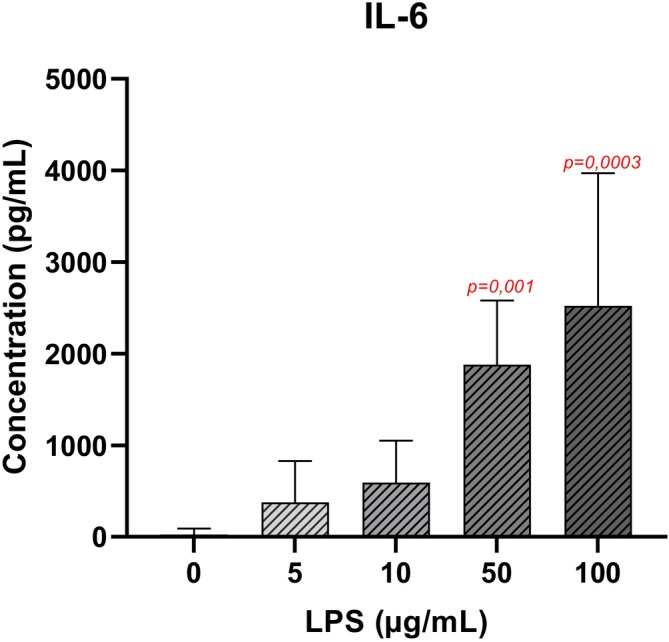
Quantification of IL‐6 by ELISA in supernatant of DRG cells 48 h after LPS 0 μg/mL (*n* = 3 biological replica); LPS 5 μg/mL (*n* = 3 biological replica); LPS 10 μg/mL (*n* = 3 biological replica); LPS 100 μg/mL (*n* = 3). Results are presented as means ± SD. The Kruskal–Wallis test followed by Dunn's post hoc test was used to compare the effects of each LPS concentration with the control group. *p*‐values <0.05 are displayed in red on the graphs.

Exposure to LPS at 50 and 100 μg/mL for 48 h significantly increased IL‐6 concentrations in the cell supernatants compared to the control cells. For example, after a 48 h exposure at 100 μg/mL of LPS, an increase of 100 times in IL‐6 concentration was observed (*p* < 0.001). At concentrations of 5 and 10 μg/mL of LPS, IL‐6 concentrations in cell supernatant tended to increase but without significance (Figure 3).

### 3.4 Mitochondrial respiration and energetic metabolism

#### 3.4.1 Oxidative metabolism

**FIGURE 4:**
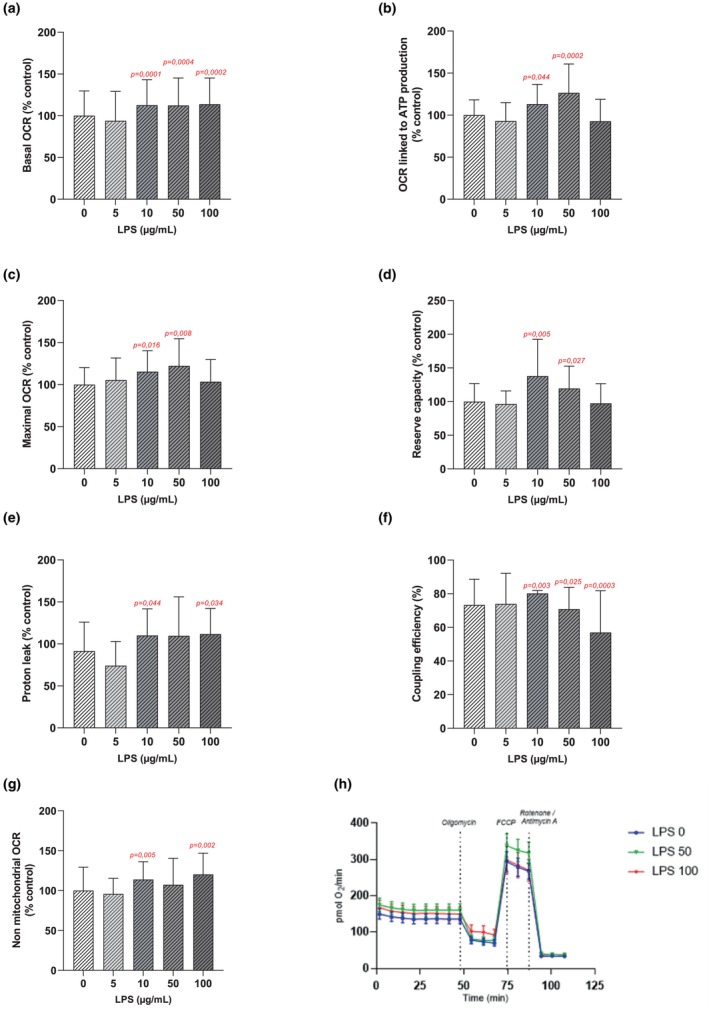
Mitochondrial respiration and energetic metabolism measured and calculated on DRG cells exposed to LPS for 48 h at 0 (*n* = 12 biological replica); 5 (*n* = 3 biological replica); 10 (*n* = 3 biological replica); 50 (*n* = 3 biological replica); 100 (*n* = 3 biological replica) μg/mL. Basal OCR (a), OCR related to ATP production (b), Maximal OCR (4c), Reserve capacity (d), Proton leak (e), Coupling efficiency (f), Non mitochondrial OCR (g); values are represented as means ± SD. Typical profiles of OCR for the concentrations of 50 μg/mL and 100 μg/mL of LPS (h); values are represented as means ± SEM. A Mann–Whitney test was used to compare each LPS concentration to the control condition. *p*‐values <0.05 are displayed in red on the graphs.

DRG cells exposure to LPS at 5 μg/mL for 48 h did not lead to any significant changes in oxidative parameters compared to the control group (Figure 4).

After 48 h of exposure to LPS, a significant increase in basal OCR was observed from 10 μg/mL (12.6 ± 3.8%; *p* = 0.0001) up to 100 μg/mL of LPS compared to the control group (Figure 4a).

Moreover, at 10 and 50 μg/mL of LPS, a significant increase in OCR linked ATP production was observed compared to the control group. For example, an increase of 13.1 ± 5.7% (*p* = 0.042) and 24.8 ± 6.6% (*p* < 0.0001) was observed after 48 h of exposure to 10 and 50 μg/mL, respectively (Figure 4b). These effects were no longer observed at 100 μg/mL of LPS. Similar results were observed for maximal OCR and reserve capacity (Figure 4c,d).

Proton leak was significantly increased after 48 h exposure to LPS at 10 μg/mL and 100 μg/mL of LPS compared to the control cells. Indeed, 10 μg/mL of LPS induced an increase of 10.1 ± 7.7% in proton leak (*p* = 0.043), while 100 μg/mL of LPS increased it by 11.6 ± 8.1% (*p* = 0.037) (Figure 4e). Similar results were noted for non‐mitochondrial OCR (Figure 4g).

Regarding the coupling efficiency of DRG cells, a 48 h exposure to LPS at 10 μg/mL significantly increased it, whereas higher concentrations of LPS (50 μg/mL and 100 μg/mL) decreased it. For example, an increase of 6.9 ± 0.4% in coupling efficiency was measured after 48 h of exposure to 10 μg/mL of LPS (*p* = 0.003) while a decrease of 16.4 ± 6.4% was observed at 100 μg/mL of LPS (*p* = 0.0003) (Figure 4f).

#### 3.4.2 Glycolytic metabolism

**FIGURE 5:**
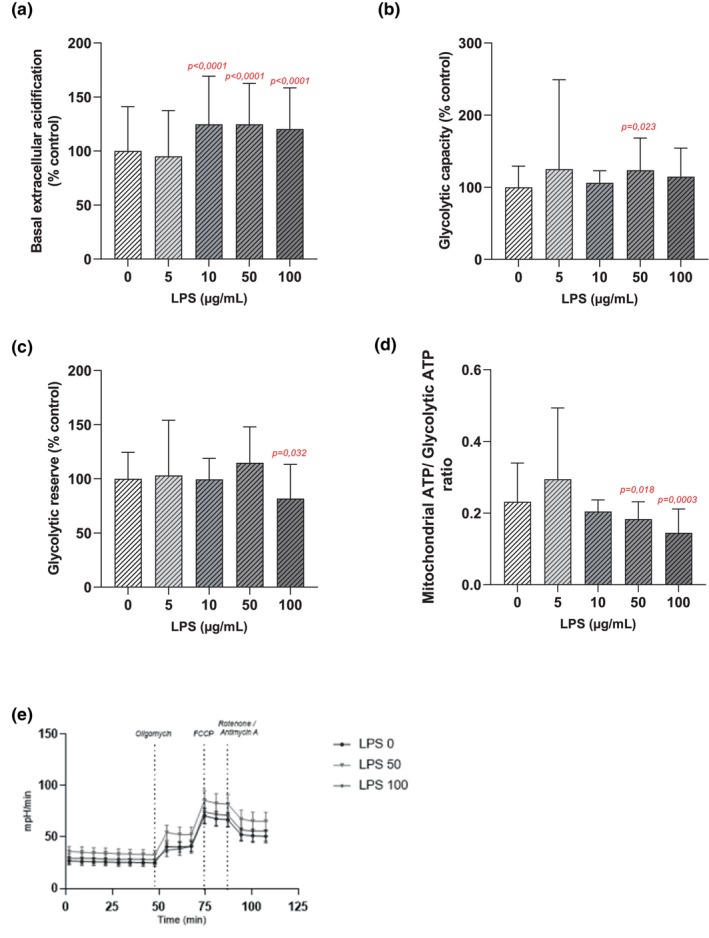
Parameters of glycolytic metabolism measured and calculated on DRG cells exposed to LPS for 48 h at 0 (*n* = 12 biological replica); 5 (*n* = 3 biological replica); 10 (*n* = 3 biological replica); 50 (*n* = 3 biological replica); 100 (*n* = 3 biological replica) μg/mL. Basal extracellular acidification (a), Glycolytic capacity (b), Glycolytic reserve (c); mitochondrial ATP/Glycolytic ATP ratio (d); values are represented as means ± SD. Typical profiles obtained for the concentrations of 50 μg/mL and 100 μg/mL of LPS (e); values are represented as means ± SEM. A Mann–Whitney test was used to compare each LPS concentration to the control condition *p*‐values <0.05 are displayed in red on the graphs.

An exposure to LPS at 5 μg/mL for 48 h did not lead to any significant changes in glycolytic parameters compared to the control group (Figure 5).

After 48 h of exposure to LPS, a significant increase in basal extracellular acidification was observed from 10 μg/mL up to 100 μg/mL of LPS compared to control cells (Figure 5a). For example, 10 μg/mL of LPS induced an increase of 24.7 ± 5.6% (*p* < 0.0001) in basal extracellular acidification and of 20.3 ± 5.0% at 100 μg/mL of LPS (*p* < 0.0001) (Figure 5a).

An exposure to 50 μg/mL of LPS for 48 h led to a significant increase of 23.6 ± 10.8% (*p* = 0.023) in glycolytic capacity (Figure 5b). Moreover, the glycolytic reserve decreased significantly by 18.2 ± 8.1% after 48 h of exposure to 100 μg/mL of LPS (*p* = 0.032) (Figure 5c).

At 50 and 100 μg/mL of LPS for 48 h, the ratio between the mitochondrial ATP and the glycolytic ATP decreased significantly by 20.9% (*p* = 0.012) and 37.5% (*p* < 0.001), respectively, compared to the control group (Figure 5d).

#### 3.4.3 Substrate utilization

**FIGURE 6:**
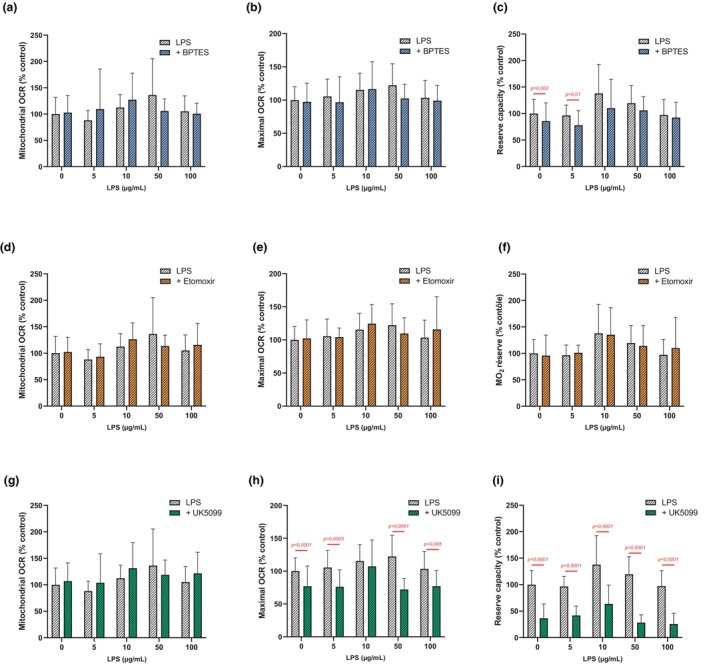
Parameters of OCR measured on DRG cells exposed to LPS for 48 h in the presence of an amino acid pathway inhibitor: Mitochondrial OCR (a), Maximal OCR (b), Reserve capacity (c) or in the presence of an fatty acid pathway inhibitor (Etomoxir): Mitochondrial OCR (d), Maximal OCR (e), Reserve capacity (f) or in the presence of a glycolysis pathway inhibitor (UK 5099): Mitochondrial OCR (g), Maximal OCR (h), Reserve capacity (i). Values are represented as means ± SD. A Mann–Whitney test was used to compare each LPS concentration to the corresponding condition without inhibitor. *p*‐values <0.05 are displayed in red on the graphs.

The inhibition of the amino acid pathway by BPTES did not lead to significant effects on the variations of mitochondrial OCR and maximal OCR induced by all concentrations of LPS (Figure 6a,b). However, without LPS exposure and at 5 μg/mL of LPS, the inhibition of the amino acid pathways induced a significant decrease in reserve capacity (respectively *p* = 0.002; *p* = 0.010) (Figure 6c). These effects of amino acid pathway inhibition on reserve capacity were no longer observed at higher LPS concentrations (10, 50 and 100 μg/mL) (Figure 6c). There was a high variability of the data of maximal OCR and reserve capacity (Figure 6b,c).

In the control condition, without LPS, the inhibition of the fatty acid pathway by etomoxir did not significantly change the mitochondrial and maximal OCR as the reserve capacity (Figure 6d–f). For all experimental conditions, the inhibition of the fatty acid pathway by etomoxir did not compensate for the changes induced by LPS (Figure 6d–f).

Moreover, for all experimental conditions, the inhibition of the glycolysis pathway by UK5099 did not have a significant effect on the variations of mitochondrial OCR (Figure 6g).

Except at 10 μg/mL of LPS, the inhibition of the glycolysis pathway led to a significant decrease in maximal OCR (Figure 6h). For example, in the absence of LPS, maximal OCR decreased by 23.0 ± 4.0% (*p* < 0.0001) when the glycolysis pathway was inhibited (Figure 6h). Furthermore, this inhibition induced a decrease of 29.4 ± 6.6% in maximal OCR at 5 μg/mL of LPS (*p* < 0.001) and by 8.1 ± 10.4% at 50 μg/mL of LPS (*p* < 0.0001) (Figure 6h). Finally, for all experimental conditions, the inhibition of the glycolysis pathway led to a significant decrease in reserve capacity (Figure 6i). Indeed, at 50 μg/mL of LPS, this inhibition decreased maximal OCR by 91.3 ± 3.9% (*p* < 0.0001) (Figure 6i). High variability of the data was observed at 10 μg/mL of LPS with or without UK5099.

## 4 DISCUSSION

In the context of sepsis, when an uncontrolled systemic inflammatory response to infection can alter the activities of tissues and organs, LPS was used to induce a direct activation of inflammatory intracellular pathways to investigate the resulting mitochondrial dysfunction. To validate our inflammatory model, we examined *IL‐6* transcripts and protein expression, as IL‐6 is a cytokine produced during NF‐κB pathway activation by various factors, including LPS. In our experiments, the overexpression of *IL‐6* transcripts and the increase in IL‐6 concentrations in cell supernatant support the validation of our neuroinflammation model. In our model, as in other studies (Andreassen et al., 2015; Nürnberger et al., 2021), LPS triggered inflammatory signaling pathways and cytokine secretions. In primary DRG cell culture, it was described that these pro‐inflammatory factors are produced by microglial cells such as macrophages (Ji et al., 2018; Kettenmann et al., 2011). These macrophages are highly important for the formation of pro‐inflammatory cytokines in response to LPS. They are crucial and represent the first line of defense against pathogens (Leisengang et al., 2018). Our results suggest an inflammatory response in DRG cells, likely leading to oxidative stress due to an imbalance between radical defense systems and reactive oxygen species (ROS) production. The tendency to *SOD2* mRNA overexpression after 48 h of LPS exposure supports this hypothesis and the establishment of oxidative stress, likely due to inflammation and secretion of cytokines like IL‐6. The same results (overexpression of *IL‐6* and *SOD2*) were observed in a neuroinflammation model (BV2 cells) exposed to 200 ng/mL of LPS for 5.5 h (Park et al., 2023).

Moreover, we investigated *LIF* transcripts, another cytokine from the IL‐6 family that shares some signaling pathways with IL‐6 and may be induced by LPS. Our study demonstrated an overexpression of *LIF* transcripts which can be beneficial as described in other studies (Alexander et al., 1992; Miyamoto et al., 2008; Sempowski et al., 2002). Indeed, some studies have demonstrated that LIF stimulated neural development and improved cell survival particularly of neuronal and Schwann cells in DRG cells (Dowsing et al., 1999; Murphy et al., 1997). Nevertheless, there are currently too few studies on LIF and its effects on DRG cells to conclude. The role of LIF may partially explain the cellular viability improvement of DRG cells exposed to 10 μg/mL of LPS for 48 h. This may also be due to the proliferation of non‐neuronal DRG cells such as immune or glial cell types also present in DRG cells extracts. In fact, LPS induced NFkB pathway activation which plays a critical role in survival and proliferation pathways, such as MAPK/ERK and PI3k/AKT (Bai et al., 2023).

Pro‐inflammatory and oxidative environments lead to cellular damage, including mitochondrial dysfunction. To maintain functional integrity and energy needs, damaged mitochondria are removed through quality control mechanisms, including mitochondrial fission and fusion. Thus, we have studied the expression of fission (*Fis1* and *Drp1*) and fusion genes (*OPA1*, *Mfn1*, and *Mfn2*). Indeed, literature indicates that LPS can increase mitochondrial fission mechanisms to contain mitochondrial damage and decrease fusion to maintain equilibrium between these processes. For example, in a murine cardiac cell model (HL‐1), LPS (10 mg/mL) significantly increased fission gene expression (*Drp1* and *Fis1*) and decreased fusion gene expression (*Mfn2* and *OPA1*) (Zhao et al., 2024). Additionally, LPS exposure in macrophages induced oxidative damage and decreased OPA1 mRNA expression while upregulating Drp1 (Shi et al., 2019). In our study, trends of *Fis 1*, *OPA 1*, and *Mfn 2* under expression after 48 h exposure to 10–100 μg/mL of LPS were observed. Further experiments are required to confirm these trends. In our study, the gene expression of subunits of various mitochondrial transport chain complexes was also investigated. Trends toward under expression of *mt‐ND1*, *mt‐Cox2*, and *mt‐ATP6* transcripts were observed after 48 h exposure at 10–100 μg/mL of LPS. As in our study, in an in vitro model of acute kidney injury induced by 4 μg/mL of LPS (HK‐2 cells), a decrease in *mt‐ND1* mRNA expression was demonstrated (Dai et al., 2019). In a chicken intestinal injury model induced by LPS challenge, decreased transcripts of *mt‐ND1*, *mt‐COX1*, and *mt‐ATP6* were also shown (Sun et al., 2020). Together, the under expression of mitochondrial genes in our study suggests some metabolic alterations and mitochondrial dysfunctions.

In our study, an increase in basal OCR for cells exposed to 10 and 50 μg/mL of LPS was observed. This suggests that LPS leads to an increase in the basal metabolic needs necessary to maintain essential cellular functions. The increase in maximal OCR at 10and 50 μg/mL of LPS indicates an improved efficiency of the electron transport chain and enhanced oxidative metabolism. The enhanced reserve capacity at these concentrations of LPS also suggests effective mitochondria that are potentially capable of adapting to metabolic disturbances. Moreover, to meet higher energy demands, an increase in OCR linked ATP production was observed. LPS, at 10 and 50 μg/mL, induced an increase in oxidative metabolism. Similar results were observed in a model of synovial fibroblasts from rheumatoid arthritis patients exposed to 1 μg/mL of LPS for 24 h (Bai et al., 2022) or in a model of primary neutrophils extracted from healthy volunteers and exposed to 0.1 μg/mL of LPS for 4 or 8 h (Pan et al., 2022). These observations may reflect a phenomenon of mitohormesis, whereby moderate exposure to mitochondrial stress induces the establishment of protective adaptive responses beneficial to the cell (Mattson & Leak, 2024; Wang et al., 2018). This phenomenon may involve Nrf2, a transcription factor that plays a critical role in mitochondrial homeostasis regulation (Gureev et al., 2019). In a Nrf2 null mice model, some severe mitochondrial dysfunctions were observed as well as an impairment of mitochondrial biogenesis (Athale et al., 2012; Joe et al., 2015). In contrast, the overexpression of Nrf2 attenuated abnormalities in mitochondrial function and protected against damages induced by LPS (Jian et al., 2022).

Furthermore, in our study, these adaptations to LPS seem to be exceeded at 100 μg/mL of LPS, potentially causing cellular and mitochondrial damage. Indeed, an increase in non‐mitochondrial OCR was observed which can reflect an increase in oxidative stress within the DRG due to the NADPH oxidase. These observations may be associated with a decrease in Nrf2 expression after LPS exposure as described in a bovine mammary epithelial cells model exposed to LPS for 12 h (Meng et al., 2022). Moreover, our study demonstrates that exposure to 10 and 100 μg/mL of LPS leads to increased proton leak. It may be indicative of mitochondrial damage as it is associated with a decrease in ATP concentration and mitochondrial membrane potential. However, by decreasing mitochondrial membrane potential, it can also be beneficial and regulate ROS production. Indeed, proton leak can be induced by UCPs 1–3, which are directly activated by SOD to induce mitochondrial membrane depolarization and reduce ROS production (Mailloux & Harper, 2011). Thus, an increase in proton leak could be a beneficial change and may allow neuroprotection. However, in our model, further investigations are needed to determine if the increase is beneficial or deleterious. In our study, we also described an increase in coupling efficiency at 10 μg/mL of LPS: mitochondria appear to use the proton gradient more efficiently to produce ATP, thereby increasing their OCR. In contrast, a decrease in coupling efficiency was observed at 50 and 100 μg/mL of LPS. The uncoupling observed may be linked to the partial opening of the mitochondrial permeability transition pore (mPTP), resulting in mitochondrial membrane potential loss (Halestrap, 2009; Halestrap et al., 2002). Ultimately, this uncoupling can lead to reduced ATP levels and cell death (Ruiz‐Ramírez et al., 2016). These results support the hypothesis of mitohormesis.

Our results seem to support LPS‐induced metabolic reprogramming. Indeed, the ratio determining the contribution of oxidative phosphorylation and glycolysis to ATP production decreased at 50 and 100 μg/mL of LPS, suggesting metabolic reprogramming toward a glycolytic phenotype in DRG cells. Similar results were found in an inflammatory model of microglial (BV2) cells, showing that LPS induced the activation of microglial cells which quickly leads to a higher use of glycolysis (Cheng et al., 2021). This metabolic reprogramming involves multiple pathways, including nitric oxide generation by iNOS, which suppresses oxidative phosphorylation, activates the mTOR pathway, increases glucose uptake, and inhibits AMPK (Kelly & O'Neill, 2015; Krawczyk et al., 2010). Moreover, exposure to the highest concentration of LPS (100 μg/mL) results in a decrease in glycolytic metabolism, as evidenced by a reduced glycolytic reserve. This suggests reduced metabolic flexibility, likely due to LPS‐induced cellular damage and impaired glycolytic function.

Our results confirm that DRG cells use glucose as a primary substrate in case of high energy demand. Indeed, when glycolysis is inhibited, the control cells and those exposed to LPS can no longer meet the high metabolic needs. Similar findings are noted for the reserve capacity, which reflects the ability of mitochondria to respond to increased energy demands. Glycolysis, which provides reduced equivalents for oxidative phosphorylation, seems essential during significant metabolic needs, and its inhibition does not appear to be compensated by amino acid or fatty acid pathways. Glycolysis allows for rapid ATP generation, thus providing immediate energy through the action of glycolytic enzymes, which can be crucial in response to stress, unlike oxidative phosphorylation, which is a slower and more complex process (Van Wyngene et al., 2018). Moreover, glycolysis produces metabolic intermediates that are essential for various cellular functions, including the synthesis of fatty acids, amino acids, and neurotransmitters (Li et al., 2023; Trigo et al., 2022). In a context of normal energy demand, the inhibition of the glutamine, fatty acid, or glycolytic pathways does not lead to changes in mitochondrial OCR, suggesting compensation by the remaining pathways. It is interesting to note that the inhibition of the amino acid pathway leads to a decrease in the reserve capacity in the absence of LPS or after an exposure at 5 μg/mL whereas these effects are no longer observed at higher LPS concentrations. Under our experimental conditions, amino acid pathways appear to be important, likely because this pathway enables cellular adaptation to energy demands during stress or glucose deprivation. Indeed, this pathway can supply energy to neurons by generating metabolic intermediates that enter into the Krebs cycle.

Cells extracted from DRG represent a heterogeneous population, predominantly composed of neurons in primary culture from embryonic rat dorsal root ganglia (Owen and Egerton, 2012). This population also includes supportive cells such as Schwann and glial cells, macrophages, and fibroblasts, making it important to consider the integrated metabolic response of the entire cell population (Haberberger et al., 2023). Nonetheless, as rightly pointed out, in cultures derived from adult rat dorsal root ganglia, satellite glial cells have been reported to represent the predominant cell type (Leisengang et al., 2018).

In conclusion, in our study, we have described the direct metabolic (mitochondrial and glycolytic) effects of LPS on activity in DRG cells that vary based on concentration. A phenomenon of mitohormesis occurs in response to LPS exposure, where DRG cells develop adaptive mechanisms to combat the stress caused by LPS. This is reflected in increased oxidative and glycolytic metabolism. However, when these adaptive mechanisms are overwhelmed, mitochondrial and cellular damage appears, leading to a potential decrease in metabolism. In the context of neuroinflammation, it would be relevant to further investigate neuro‐metabolic disturbances by combining the direct effects of LPS with proinflammatory cytokines that simulate systemic inflammation. This would help to better understand the combined impact of LPS and systemic inflammatory signals on cellular metabolism. In addition, it would be interesting to explore the role of TRP channels, as they are known to play a key role in the regulation of inflammation through sensory functions and the release of neuropeptides (Silverman et al., 2020).

The most notable limitation of this study is the lack of in vivo experiments. Our findings are obtained from in vitro experiments and may not fully describe the complexity and integrated responses of living organisms. While our in vitro approaches allow us to describe informative mechanisms, they cannot account for systemic interactions. Therefore, further validation in in vivo models is needed.

## FUNDING INFORMATION

No funding was received.

## CONFLICT OF INTEREST STATEMENT

The authors have no conflict of interest to declare.

## ETHICS STATEMENT

This research did not require ethics approval because it was conducted using established cell lines.

## Data Availability

The data that support the findings of this study are available on request from the corresponding author.
